# The Role of Vitamin D in Disease Progression in Early Parkinson’s Disease

**DOI:** 10.3233/JPD-171122

**Published:** 2017-11-01

**Authors:** Isobel Sleeman, Terry Aspray, Rachael Lawson, Shirley Coleman, Gordon Duncan, Tien K. Khoo, Inez Schoenmakers, Lynn Rochester, David Burn, Alison Yarnall

**Affiliations:** a Clinical Ageing Research Unit, Newcastle University, Campus for Ageing and Vitality, Newcastle upon Tyne, UK; b Bone Clinic, Freeman Hospital, Freeman Road, Newcastle upon Tyne, UK; c Industrial Statistics Research Unit, Herschel Building, Newcastle University, Newcastle upon Tyne, UK; d Department of Geriatric Medicine, University of Edinburgh, Edinburgh, UK; e School of Medicine and Menzies Health Institute Queensland, Griffith University, QLD, Australia; f Department of Medicine, Faculty of Medicine and Health Sciences, University of East Anglia, Norwich Research Park, Norwich, UK; g MRC Human Nutrition Research, Cambridge, UK; h Institute of Neuroscience, Newcastle University, Clinical Ageing Research Unit, Campus for Ageing and Vitality, UK; i Institute of Neuroscience, The Medical School, Newcastle University, UK

**Keywords:** Balance, cognition, disease progression, fall, 25-hydroxy vitamin D, Parkinson’s disease, vitamin D

## Abstract

**Background:**

Previous cross-sectional studies have shown that Parkinson’s disease (PD) patients have lower serum 25-hydroxy vitamin D (25(OH)D) concentrations than controls. Vitamin D deficiency was associated with increased disease severity and cognitive impairment in prevalent PD patients.

**Objective:**

The aim of the study was to determine 25(OH)D in newly diagnosed PD and age-matched controls and to assess if there was an association with clinical outcomes (disease severity, cognition and falls) over the 36-month follow up period.

**Methods:**

A prospective observational study of newly diagnosed PD patients in the North East of England with age-matched controls (PD, *n* = 145; control, *n* = 94). Serum 25(OH)D was assessed at baseline and 18 months. Participants underwent clinical assessment at baseline, 18 and 36 months. One hundred and ten participants with PD also took part in a prospective falls study.

**Results:**

Mean serum 25(OH)D concentrations were lower in PD than control participants at baseline (44.1±21.7 vs. 52.2±22.1 nmol/L, *p* < 0.05) and 18 months (44.2±23.6 vs. 55.7±28.8 nmol/L, *p* < 0.05). Baseline serum 25(OH)D concentration, age, motor score and dosage of dopaminergic medication were significant predictors of variance of motor severity at 36 months ((*Δ*R^2^ = 0.039, F = 6.6, *p* < 0.01). Serum 25(OH)D was not associated with cognition or falls during the follow up period.

**Conclusions:**

Patients with incident PD had significantly lower serum 25(OH)D concentrations than age-matched controls, which may have implications in terms of bone health and fracture risk. There was a small but significant association between vitamin D status at baseline and disease motor severity at 36 months.

## INTRODUCTION

Idiopathic Parkinson’s disease (PD) is the second most common neurodegenerative disorder in the United Kingdom (UK) after Alzheimer’s disease, with increasing age being the greatest risk factor for the development of the disorder. Thus, any interventions with the potential to reduce the incidence of the disease or its complications receive considerable attention.

Outdoor work has been associated with a reduced risk of developing PD in later life. Danish men who worked outdoors were less likely to develop PD than men who worked indoors (odds ratio 0.72 (95% CI 0.63 to 0.82)) [1]. One possible mechanism for the protective effect of outdoor work may be increased exposure to sunlight, which leads to cutaneous vitamin D synthesis. This hypothesis is supported by a Finnish study, which showed that middle-aged people with a serum 25-hydroxy vitamin D (25(OH)D) concentration in the highest quartile were a third less likely to develop PD than those in the bottom quartile [2]. A recent study in France used population data to show that areas with higher UV-B radiation had lower rates of prescription of dopaminergic medications in the under 70 s, suggesting lower rates of PD [3]. This is the first data looking at the link between sunlight exposure and risk of PD, however, the estimated sunlight exposure may vary significantly between individuals. Conversely, a recent study in the United States has failed to confirm the association between mid-life vitamin D status and prospective risk of PD [4]. This may be related to the higher population vitamin D levels in the US compared to Finland. These findings suggest that an increased risk may be particularly found amongst those with a very low vitamin D status, also common in the UK. Therefore, further studies in well-designed cohorts are required.

As postmortem studies have shown that there are vitamin D receptors on dopaminergic neurons in the human substantia nigra, it has been suggested that vitamin D may be able to protect dopaminergic neurons [5]. Whilst this has not been directly proven in humans, the administration of vitamin D in animal models of PD were found to be neuroprotective to dopaminergic neurons [6].

Cross-sectional studies have demonstrated that patients with established PD have significantly lower plasma concentrations of 25(OH)D than age-matched controls [7, 8]. Vitamin D deficiency has been correlated with increasing motor severity, postural instability [9] as well as poorer verbal fluency and memory [10]. It is unclear if these effects are specific to PD, as vitamin D deficiency is also associated with postural instability [11] and cognitive impairment [12] in community dwelling older adults. While these studies of the general older population have been large and well designed, the studies of PD participants have small numbers and cross-sectional design, making it difficult to determine if vitamin D status is an independent risk factor, or a marker of disease severity.

We therefore sought to determine vitamin D status in a longitudinal cohort of incident PD subjects and age-matched controls at time of PD diagnosis and 18 months later. Secondary outcome measures included associations between vitamin D status and cognition, motor severity and time to first fall among participants with PD over 36 months follow-up.

## METHODS

### Participants

Patients with newly diagnosed PD in Newcastle upon Tyne and Gateshead, England (latitude 55°North) were recruited to the Incidence of Cognitive Impairment in Cohorts with Longitudinal Evaluation - Parkinson’s disease (ICICLE-PD) study between June 2009 and December 2011, as previously described [13, 14]. PD participants fulfilled the UK Brain Bank Criteria for idiopathic PD [15]. Exclusion criteria were: significant memory impairment at baseline (defined by Mini-Mental State Score (MMSE)<24 or fulfilling the Movement Disorder Society PD dementia (PDD) criteria [16]; Lewy body dementia; insufficient English to complete neuropsychological assessments; or a diagnosis of atypical, vascular or drug-induced Parkinsonism.

Healthy control subjects were recruited locally by advertising, word of mouth and community groups. Spouses, relatives and carers were not eligible to limit bias. Control subjects were excluded if they had a major psychiatric disorder, cognitive impairment, previous stroke, or movement disorder.

All of the control participants were Caucasian (*n* = 94). One of the PD participants (*n* = 145) was African; three were Asian and the remainderCaucasian.

### Ethics

The study was approved by Newcastle and North Tyneside Research Ethics Committee. All participants provided written informed consent.

### Clinical assessment

All subjects underwent a clinical assessment at baseline, 18 and 36 months. Each assessment included a clinical history including comorbidities, current medication use and social history, and a clinical examination by a movement disorder specialist. The PD group also underwent assessment of their disease using the Movement Disorder Society Unified Parkinson’s Disease Rating Scale (MDS-UPDRS) parts II and III [17], and Hoehn and Yahr scale [18]. Mood was assessed using the 15-point Geriatric Depression Scale [19]. Cognition was assessed using the MMSE [20], and two batteries of computerised tests – the Cognitive Drug Research (CDR) battery [21] and Cambridge Neuropsychological Test Automated Battery (CANTAB) [22], as described previously [14]. A diagnosis of mild cognitive impairment in PD (PD-MCI) was based on a modified version of the criteria defined by the Movement Disorder Society taskforce, in common with other ICICLE publications, using two standard deviations as the cut off [23]. Motor and cognitive testing was performed with participants having taken their usual medication.

A subset of consenting participants (*n* = 110) with PD completed monthly prospective falls diaries for 36 months as part of a nested study (ICICLE-GAIT). A fall was defined as an event that causes a person to unintentionally come to rest on the ground or another lower level [24]. Participants were asked to record if they had fallen in the previous month and to give the date, time, location and circumstances of any falls. Study staff called participants to verify the details of any reported falls and to seek clarification if required. Falls study participants did not differ from the whole group with respect to age, gender, disease duration and severity at baseline (data not shown).

### Vitamin D analysis

Serum 25(OH)D levels were determined at baseline and 18 months. Blood samples were drawn while fasting. Samples were centrifuged (4000 rotations per minute for 10 minutes at 19°C), and serum was transferred into cryovials and stored at –80°C. Samples were transferred to the MRC Human Nutrition Research (HNR) in Cambridge on dry ice for analysis. They were analysed individually by liquid chromatography tandem mass spectrometry (LC-MSMS) to determine 25(OH)D_2_ and 25(OH)D_3_ concentration. Total 25(OH)D concentration wasdetermined by summing 25(OH)D_2_ and 25(OH)D_3_ concentration. The HNR laboratory is a member of the Vitamin D Standardisation Programme (VDSP), and quality assurance of 25(OH)D assays are performed as part of the Vitamin D External Quality Assessment Scheme (www.deqas.org). Assay performance was monitored using Chromsystems and in-house controls. The inter assay variation was <10% for 25(OH)D_2_ and <7% for 25(OH)D_3_ and the limit of quantification was 6nmol/L.

### Statistical analysis

Data were analysed using IBM SPSS 22. They were checked for normality by visual inspection of histograms and the Kolmogorov-Smirnov test and compared using independent *t*-tests or Mann-Whitney U tests, depending on distribution. The chi-square test was used to assess binary outcome variables. The significance level for all statistical tests was set at 0.05 two-tailed.

As serum samples were drawn throughout the year, serum 25(OH)D concentration varied by season in both the PD patients and controls, which made comparison between individuals unfeasible. Control data for baseline and 18 months was therefore pooled and a standard curve derived (See Supplementary Figure 1). Data for individuals was then compared to the mean, difference from the mean was calculated to give an adjusted serum 25(OH)D value at baseline. This value was then used in the models described below. Effect size was determined using eta-squared coefficients.

Multiple regression was performed to determine significant predictors of disease severity using MDS-UPDRS part III score at 36 months. Preliminary analyses were conducted to ensure no violations of the assumptions of normality, linearity, multicollinearity and homoscedasticity. Predictors of MDS-UPDRS part III score at 36 months comprised baseline age, MDS-UPDRS part III score and levodopa equivalent dose (LED). Serum 25(OH)D concentration, adjusted as described above, was then added to the model.

Logistic regression was used to predict cognition at 36 months using PD-MCI classification as the dependent variable. Based on previous work, covariates entered into the models included baseline age and years of education as predictors of cognition [14].

Falls data was dichotomised to compare participants who were above and below the threshold for vitamin D deficiency (50 nmol/L 25(OH)D). Time to first fall was plotted as a Kaplan-Meier curve and survival between groups compared using the log rank test. Cox regression was used to determine if vitamin D deficiency was associated with shorter time to first fall, after adjusting for risk factors, such as previous falls, disease severity, and MMSE score [25].

## RESULTS

### Baseline characteristics

At baseline, 145 PD and 94 control participants were included (see Flowchart; Supplementary Figure 2). Participant baseline characteristics are shown in Table 1. PD and control groups were well matched for age. As previously described [14], subjects with PD demonstrated impairments on measures of cognition and mood compared to the control group.

**Table 1:** Characteristics of participants at baseline (data are mean (SD) unless stated)

	Control participants	PD participants	*P* value
	N = 94	N = 145
Age (years)	68.2 (8.1)	66.2 (11.7)	0.41^a^
Male, n (%)	50 (53.2)	94 (64.8)	0.09^b^
Height (m)	1.7 (0.1)	1.7 (0.2)	0.53^c^
Weight (kg)	80.2 (14.2)	79.0 (16.3)	0.55^c^
Disease duration (months)	n/a	6.2 (6.1)	n/a
LEDD (mg/day)	n/a	168.8 (129.8)	n/a
Hoehn and Yahr stage	n/a	2.0 (0.7)	n/a
MDS-UPDRS Part II	n/a	10.9 (5.8)	n/a
MDS-UPDRS Part III	n/a	26.6 (11.9)	n/a
MMSE score	29.0 (1.2)	28.6 (1.4)	<0.01^c^
PD-MCI 2 SD, n (%)	9 (9.6)	37 (25.5)	0.002^b^
GDS-15	1.0 (1.6)	2.8 (2.6)	<0.001^a^
Education, years	13.1 (3.4)	12.8 (3.9)	0.29^c^
Serum 25(OH)D (nmol/L)	52.2 (22.1)	44.1 (21.7)	<0.005^c^
% vitamin D insufficient 25(OH)D<75 nmol/L	79.8%	91.0%	0.02^b^
Serum 25(OH)D deficiency (<50 nmol/L)	45.7%	66.9%	<0.01^b^
Number on vitamin D supplements, n (%)	6 (6.4%)	11 (7.6%)	1.0^b^

GDS-15: Geriatric Depression Scale score; IQR: interquartile range; LEDD; levodopa-equivalent daily dose; PD-MCI 2 SD: mild cognitive impairment with the cut-off 2 standard deviations from normal; MMSE: Mini Mental State Examination; MDS-UPDRS: Movement Disorders Society Unified Parkinson’s Disease Rating Scale. ^a^Mann-Whitney U test, ^b^Fisher’s exact test, ^c^independent *t*-test.

### Vitamin D at baseline

**Fig.1:**
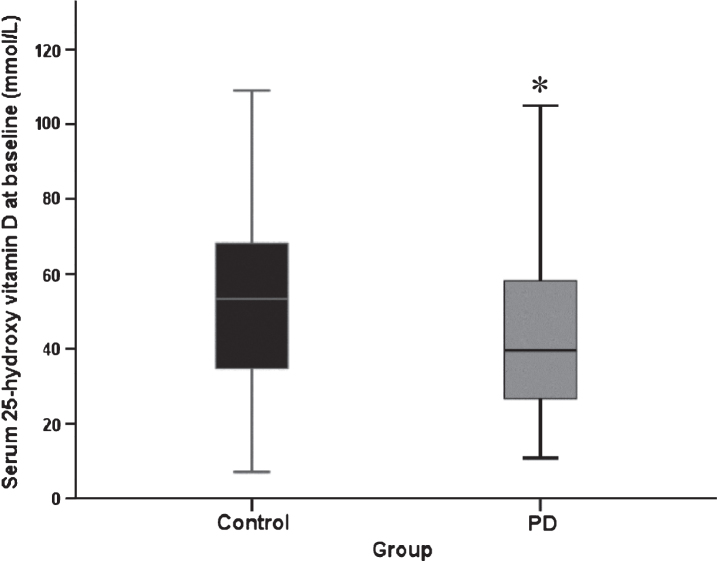
Serum 25(OH)D concentration at baseline (PD, *n* = 145, control *n* = 94. Data represent median±95% confidence interval. *p = 0.02 versus control participants (Mann Whitney U test).

Mean serum 25(OH)D concentrations were significantly lower at baseline in PD participants than controls (44.1±21.7 (mean±SD) vs. 52.2±22.1 nmol/L, respectively, *p* = 0.005, Fig. 1). Effect size was moderate (eta-squared = 0.067). Fifty-eight controls (61.7%) and 60 PD participants (41.4%) had the serum sample drawn in winter or spring (December to May), meaning that significantly more controls had a sample drawn when serum vitamin D was likely to be at its lowest (*χ*^2^ = 8.05, *p* = 0.03). There was no significant difference in serum vitamin D between those who did and did not take vitamin D supplements (*p* > 0.05 for both groups).

### Vitamin D at 18 months

At 18 months, mean serum 25(OH)D concentrations were again significantly lower among PD participants than controls (44.2±23.6 vs. 55.7±28.8nmol/L, respectively, *p* = 0.002). Controls weresignificantly more likely to have their serum sample drawn in summer or autumn (June to November). Twenty-five control subjects (32.1%) and 66 PD subjects (54.5%) had the serum sample drawn in winter or spring (*χ*^2^ = 8.54, *p* = 0.03). Eight control and 15 PD participants took vitamin D supplements at 18 months (Fisher’s exact test, *p* > 0.05 for bothgroups).

### Cognition

At 36 months, data on cognition was available for 105 PD patients and 70 controls. Participants with PD were signficantly more likely to have MCI than controls (50.5% vs. 9.7%, *χ*^2^ = 29.36, *p* < 0.001). Logistic regression revealed that baseline age, GDS score, years of education and disease group produced a reasonable model of MCI at 36 months (*χ*^2^ = 74.96, *p* < 0.001), which was not improved by the addition of baseline adjusted vitamin D status (Supplementary Table 1).

### Motor progression

We examined the relationship between vitamin D status and severity of motor impairment in participants with PD over 36 months. Mean MDS-UPDRS III score increased from 27.0±12.0 at baseline to 38.6±13.2 at 36 months. Multiple regression revealed that baseline age, MDS-UPDRS III score and LED were significant predictors of variance of motor severity at 36 months (adjusted R^2^ = 0.368, F = 19.6, *p* < 0.001), which was improved when baseline 25(OH)D was added to the model (*Δ*R^2^ = 0.039, F = 6.6, *p* < 0.01; Supplementary Table 2).

### Falls

**Fig.2:**
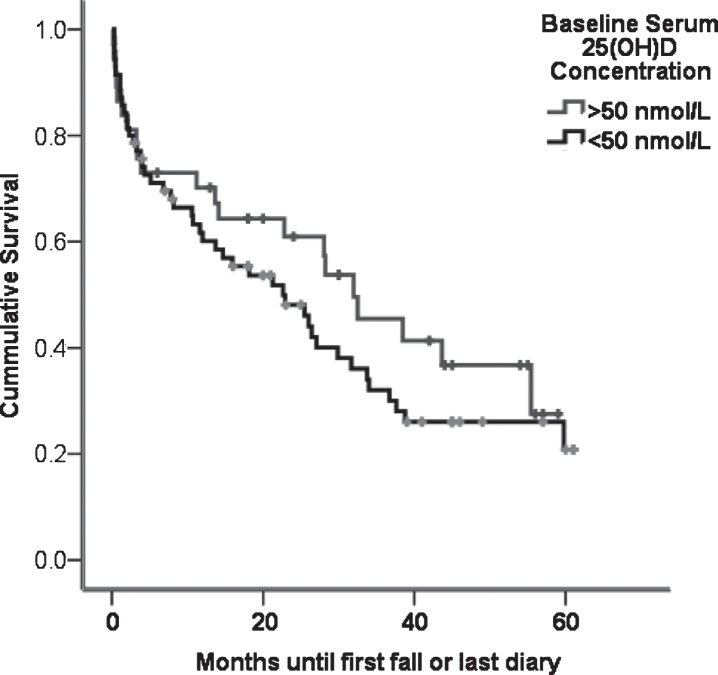
Time in months to first fall in falls diary PD participants who had above (*n* = 55) or below (*n* = 55) the median vitamin D. There was no significant difference in time-to-first-fall between the groups (log rank test, *p* = 0.23).

The median time of first fall amongst 110 PD participants who completed prospective falls diaries from baseline was 15.9 months (IQR = 1.1–30.8). There was no difference in the time to first fall between participants who were vitamin D deficient (25(OH)D<50 nmol/L) or replete (Fig. 2, log rank test, *p* = 0.32). Furthermore, Cox regression adjusting for previous falls, baseline MDS-UPDRS III and MMSE score did not reveal an increased hazard of falling among those who were vitamin D deficient (*p* = 0.30).

## DISCUSSION

We have shown that participants with early PD have lower serum concentrations of 25(OH)D than control participants within months of diagnosis, and at 18 months follow-up. Low baseline 25(OH)D concentration was associated with a higher MDS- UPDRS III score and thus greater motor severity at 36 months. No association was found between vitamin D status at baseline and time to first fall or risk of PD-MCI.

The results for the control participants are consistent with a previous study in the UK, which demonstrated that community dwelling men and women over 65 had median serum 25(OH)D concentrations of 50.5 and 43.0 nmol/L respectively [26]. In common with previous cross-sectional and longitudinal [8] studies, we found that serum 25(OH)D concentrations were significantly lower in participants with PD than controls. In our study, however, serum 25(OH)D was tested closer to the start of the disease (typically less than six months) than previously, which lends support to the hypothesis that vitamin D may have a role in disease progression. In terms of the 25(OH)D concentrations, our results were comparable to another Northern European study of patients with established disease (median duration 4.5 years) where again participants with PD had significantly lower vitamin D levels than controls (48.3 vs 56.9 nmol/L) [29].

Studies in North America have revealed much higher 25(OH)D concentrations in PD than seen in this work performed in Newcastle upon Tyne, UK (latitude 55° North). The DATATOP study, based in Atlanta, Georgia, USA (34° North), found that 69.4% of participants were vitamin D insufficient (defined as less than 75 nmol/L) at 18 months [8], compared to 91.0% in our study. Similarly, in the Harvard Biomarkers Study, based in Boston, MA, USA (42° North) mean 25(OH)D concentration was 70.0 nmol/L in a cohort of patients with a median disease duration of 4.9 years [6].

Two studies in Portland, Oregon, USA (45° North) showed associations between serum 25(OH)D and balance [9], and the risk of PD dementia [10]. Both studies reported much higher serum 25(OH)D concentrations than our study (median ∼85 nmol/L), which is likely to be related to latitude, although the wider use of supplements and food fortification in the US may have contributed. Dietary intake of vitamin D in the US is higher than in the UK (on average 7.3 versus 3.7 microgram/day [27]). It is therefore possible that the lack of association between serum 25(OH)D and functional outcomes in our study may be due to a ‘floor effect’, whereby there are no differences in function at such low 25(OH)D concentrations.

The main strengths of the study are longitudinal design, inclusion of a control group and use of the most reliable method of determining serum 25(OH)D concentration (tandem mass spectrometry) [28]. Limitations include the fact that 25(OH)D concentrations vary significantly during the year, peaking in late summer [26]. However, in this study serum samples were drawn throughout the year as participants were recruited and re-assessed at 18 months. This meant that 25(OH)D concentrations were not directly comparable between participants and may have blunted our ability to find associations between vitamin D status and clinical parameters. A small number of participants took vitamin D or multivitamin supplements during the study which may have influenced the results. However, no significant differences between those who did and did not take supplements were not found. In addition, we repeated the regression analysis excluding these participants and the models did not significantly differ (data not shown).

The main clinical significance of this work relates both to the small but significant association between baseline 25(OH)D concentration and motor severity, as assessed using MDS-UPDRS III at 36 months. In addition, this work has relevance to bone health in Parkinson’s disease. Previous research has shown a high prevalence of osteopenia and osteoporosis in cross-sectional cohorts of Parkinson’s patients, placing patients at greater risk of bone fractures should they fall. More recently, work from an incidence cohort of Parkinsonian patients and age matched controls has revealed that an increased risk of major bone fracture following a fall from diagnosis onwards (4.2% vs 1.4% per year) [29]. As vitamin D deficiency is a well-established risk factor for osteoporosis [30], our finding that the majority of newly diagnosed patients with PD are vitamin D deficient suggests that clinicians should be routinely assessing fracture risk at diagnosis and treating asappropriate [31].

## CONFLICT OF INTEREST

The authors have no conflict of interest to report.

## Supplementary Material

Supplementary Material
