# Supplementary material for: The Role of Vitamin D in Disease Progression in Early Parkinson’s Disease
Source: J Parkinsons Dis. 2017 Nov 1;7(4):669–75. doi: 10.3233/JPD-171122 (PMC5676984; doi:10.3233/JPD-171122)
Supplement: Supplementary Material [file jpd-7-jpd171122-s001.docx]

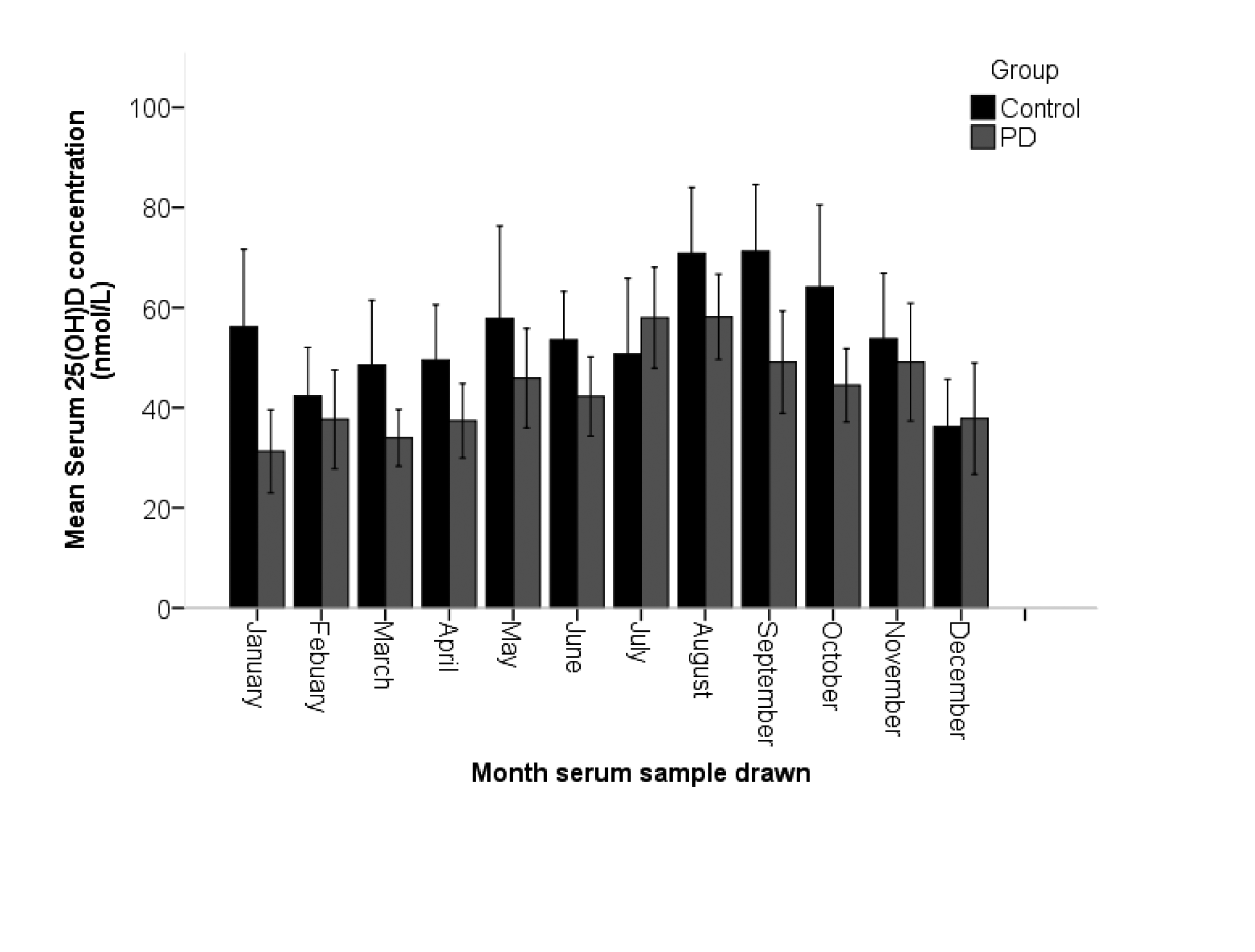


Supplementary Figure 1: Serum 25-hydroxy vitamin D concentration by month serum sample drawn


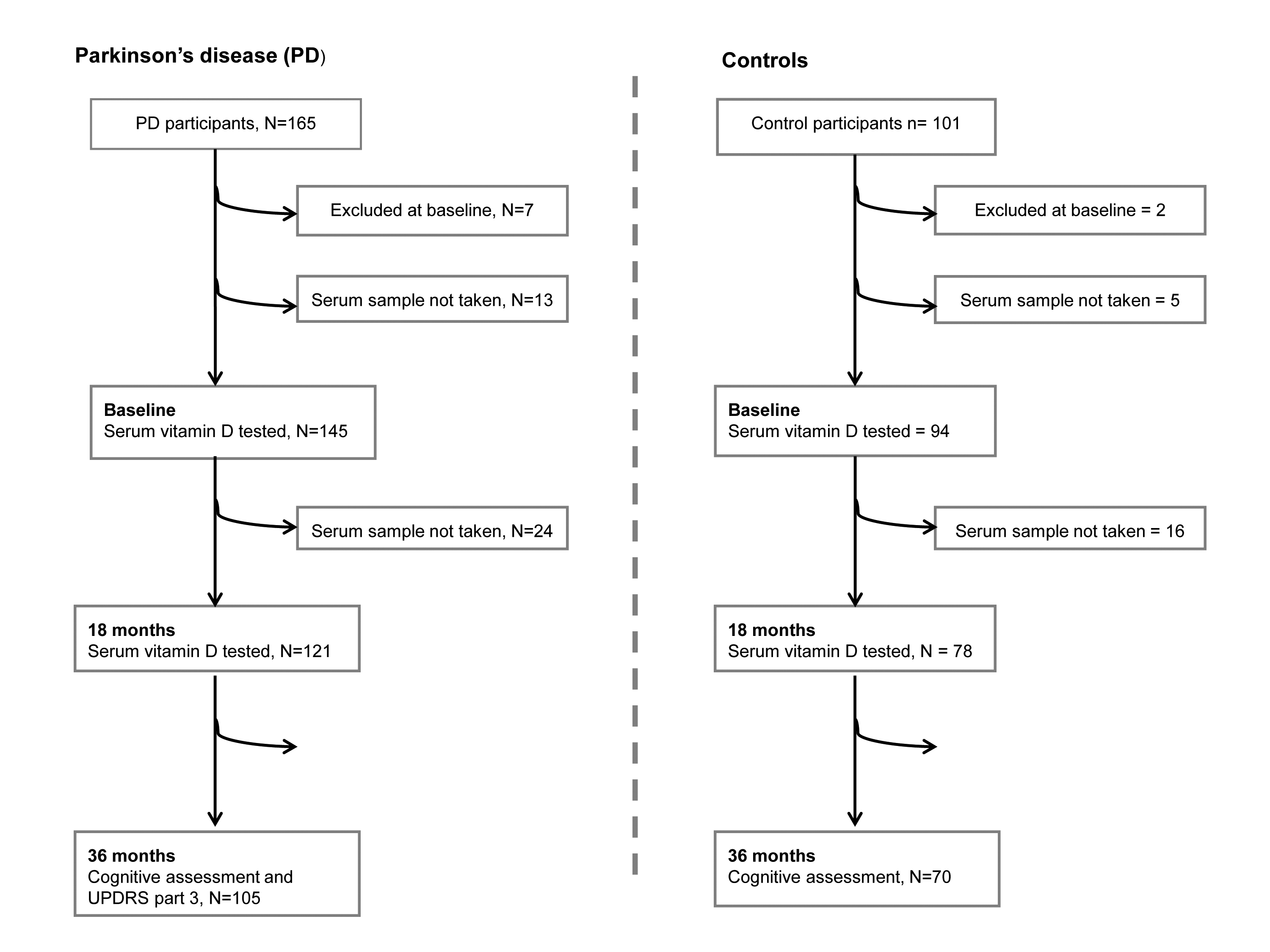


Supplementary Figure 2: Flowchart of participants

Supplementary Table 1: Binary logistic regression statistics for MCI at 36 months

|  | *B (SE)* | P-value | Exp (B) | 95% CI for Exp (B) |
| --- | --- | --- | --- | --- |
| **PD**  **Baseline age**  **Baseline GDS**  **Years education**  **Adjusted vitamin D at baseline** | 2.57 (0.55)  0.13 (0.03)  0.19 (0.09)  -0.13 (0.06)  -0.07 (0.01) | <0.001  <0.001  0.04  0.03  0.53 | 13.1  1.13  1.21  0.88  0.99 | 4.5 – 38.3  1.08 – 1.20  1.01 – 1.44  0.78 – 0.99  0.97-1.01 |
|  |  |  |  |  |
|  |  |  |  |  |

R^2^ = 0.35 (Cox & Snell), 0.49 (Nagelkerke); Model Chi-squared (5) = 75.6, p < 0.001

Supplementary Table 2: Multiple regression for UPDRS III score at 36 months

|  | β | P-value |
| --- | --- | --- |
| *Model 1* |  |  |
| UPDRS III | 0.532 | <0.001 |
| Baseline age | 0.266 | 0.001 |
| Baseline LED | -0.033 | 0.681 |
| *R^2^ = .0.363; P-value of change <0.001* | | |
|  |  |  |
| *Model 2* |  |  |
| UPDRS III | 0.523 | <0.001 |
| Baseline age | 0.268 | 0.001 |
| Baseline LED | -0.074 | 0.352 |
| Adjusted vitamin D | -0.202 | 0.012 |
| *R^2^ =0.383 ; P-value of change = 0.012* | | |
